# Prevalence of Preterm Birth in a Marginalized Roma Population—Quantitative Analysis in One of the Most Disadvantaged Regions of Hungary

**DOI:** 10.3390/ijerph22081270

**Published:** 2025-08-14

**Authors:** Kinga Pauwlik, Anita R. Fedor

**Affiliations:** 1 Doctoral School of Health Sciences, University of Pécs, Vörösmarty M. u. 4, 7621 Pécs, Hungary; 2 University of Debrecen Faculty of Health Sciences, Institute of Social Sciences, Department of Social Sciences and Social Work, Quality of Life and Sociology of Health Coordination Research Centre, University of Debrecen, 2-4. Sóstói Str., 4400 Nyíregyháza, Hungary; fedor.anita@etk.unideb.hu

**Keywords:** Roma women, preterm birth, smoking, alcohol and drug use, infection, antenatal care, health behavior, social inequalities

## Abstract

Preterm birth is a leading cause of perinatal morbidity and mortality and is particularly prevalent among socially disadvantaged female populations. This quantitative, cross-sectional study aimed to explore the prevalence of preterm birth in three segregated Roma communities in Hungary and to identify health behavior and care factors associated with its occurrence. In our study, preterm birth was defined as delivery before 37 completed weeks of gestation (i.e., <259 days). Data were collected from 231 Roma women living in three municipalities of Szabolcs-Szatmár-Bereg County, one of Hungary’s most disadvantaged regions, using a structured interview questionnaire. The participants were women aged 18–65 years. Of these, 209 had been pregnant at least once in their lifetime. The questionnaire covered socio-demographic characteristics (age, level of education, employment status, housing conditions, marital status), health behaviors (smoking, alcohol consumption, drug use, vitamin supplementation, other substance use), antenatal care attendance, and birth outcomes (preterm birth, gestational age, low birth weight, newborn status). Statistical analyses included descriptive statistics, chi-square tests, and binary logistic regression with significance set at *p* < 0.05. Preterm birth was significantly more common among women who smoked, consumed alcohol or drugs during pregnancy, or had vaginal infections. Drug use showed the strongest association with a 22-fold increase in risk, followed by alcohol (nearly fivefold), smoking (over threefold), and infections (threefold). Although non-attendance at antenatal care was associated with increased risk, this relationship was not statistically significant. In the multivariate logistic regression model, alcohol consumption (OR = 1.744, *p* < 0.01), smoking (OR = 2.495, *p* < 0.01), drug use (OR = 25.500, *p* < 0.001), and vaginal infections (OR = 4.014, *p* < 0.01) during pregnancy were independently associated with an increased risk of preterm birth, whereas folic acid supplementation (OR = 0.448, *p* < 0.05) showed a significant protective effect. These findings highlight that preterm birth is intricately linked to socioeconomic disadvantage and adverse health behaviors. Culture-specific, community-based prevention strategies are essential to reduce perinatal risks in marginalized populations.

## 1. Introduction

Premature birth is an increasingly serious public health challenge worldwide, not only affecting the chances of newborn survival, but also having a serious long-term impact on children’s physical, neurological, and mental development. In this study, preterm birth was defined according to the standard WHO definition as any birth occurring before 37 completed weeks of gestation, regardless of the infant’s size or weight [1]. This definition is internationally accepted and facilitates the comparability of the study findings with other population-based research.

According to the latest available international data, in 2020, an estimated 13.4 million newborns were born before 37 weeks of pregnancy, and of these, nearly 1 million children died due to complications related to preterm birth [2]. The latest publicly available Hungarian data on preterm birth are from the 2016 publication of the Hungarian Central Statistical Office [3,4]. According to these, 8.5% of live births in 2016 occurred before the 37th week of pregnancy, i.e., as preterm births. The proportion of low birth weight (below 2500 g) newborns was 8.5% in the same year. These rates have been relatively stable since the early 1990s, with minor fluctuations. There is a strong correlation between the prevalence of preterm births and low birth weight, but both factors alone pose a significant health risk for newborns [4]. According to revised data from the Central Statistical Office for 2023, 7.7% of live births and 8.0% of live births in 2024 are estimated to be below 2500 g; however, public statistics still do not include a breakdown by week of gestation, so it is not possible to distinguish precisely whether these cases are due to preterm birth (PTB) or intrauterine growth retardation (SGA) [5]. The underlying factors are complex, with socioeconomic conditions, maternal education, living conditions, and access to health care playing a key role, in addition to biological and medical risks. Particularly at risk are marginalized groups, such as women from ethnic minorities, for whom structural disadvantages and lifestyle factors that are detrimental to health are often cumulative [6,7,8,9,10,11]. Taking these social inequalities into account is essential when developing strategies to prevent preterm births, especially in regions where reproductive health indicators are persistently poor [12].

Two thirds of infant deaths occur in conjunction with premature births. Premature birth is a health problem but also largely a social, lifestyle, and cultural problem, occurring almost four times more frequently in very low-skilled women (with primary education or less). The life expectancy of the unborn child is significantly affected by whether the pregnancy was planned and how well prepared it was. The expectant woman’s lifestyle, social circumstances, and personal hygiene are key factors for healthy reproduction and have a direct impact on the child’s condition at birth. Research in recent years has shown that low birth weight correlates with both cardiovascular disease in adulthood and hypertension. It can therefore be concluded that reducing the rate of preterm birth is key [13].

The results of studies conducted in recent years have confirmed that low birth weight and preterm birth are significantly influenced by maternal lifestyle factors as well as by the social status of the mother [14,15,16,17,18].

A 2010 study showed that rates of preterm birth (PTB) and low birth weight (LBW) were significantly higher among Roma women in Szabolcs-Szatmár-Bereg County than among non-Roma women. The results highlighted that among Roma women, low education, smoking, poverty, and inadequate nutrition are significant factors in the prevalence of PTB and LBW [3,19]. Using older KSH data from 2007, it is also possible to detect which counties had the highest rates of LBW and/or preterm births (PTB) in Hungary. Based on the 2007 results, Szabolcs-Szatmár-Bereg County has the worst LBW rate (11.4%) and the second worst PTB rate (10.3%) [3,19].

Reproductive health indicators in Szabolcs-Szatmár-Bereg County continue to compare unfavorably with the national average. Although the most recent county-level data on the proportion of low-birth-weight newborns are not available, trends in previous years suggest that this proportion is also above the national average [20].

Maternal lifestyle has a significant impact on early childhood development, and habits during pregnancy can have a long-term impact on child health and development. Smoking increases the risk of preterm birth, low birth weight, and respiratory problems. Alcohol consumption can lead to the development of fetal alcohol spectrum disorder (FASD), which can cause long-term cognitive and physical problems. Inadequate nutrition, on the other hand, can lead to poor fetal development and increase the risk of early life diseases [21,22].

National data on the Hungarian population and birth indicators were obtained from the official reports of the Hungarian Central Statistical Office (KSH). In this study, reproductive health and perinatal outcomes were characterized based on the following indicators directly related to the main research question: number of pregnancies and births, occurrence of preterm birth and low birth weight, gestational week at delivery, maternal smoking and alcohol consumption during pregnancy, antenatal care attendance, and pregnancy-related diseases and obstetric complications.

These statistics and the literature suggest that Hungary needs to continue to pay close attention to early childhood health care and the development of antenatal care in order to catch up with the EU average.

The aim of this study was to determine the prevalence of preterm birth among marginalized Roma women, to explore the associations between preterm birth and sociodemographic characteristics, health behaviors, and reproductive health indicators, and to lay the groundwork for a comprehensive prevention program, grounded in health sociology and multidisciplinary scientific principles, which may contribute to improving perinatal outcomes.

## 2. Materials and Methods

**Figure 1:**
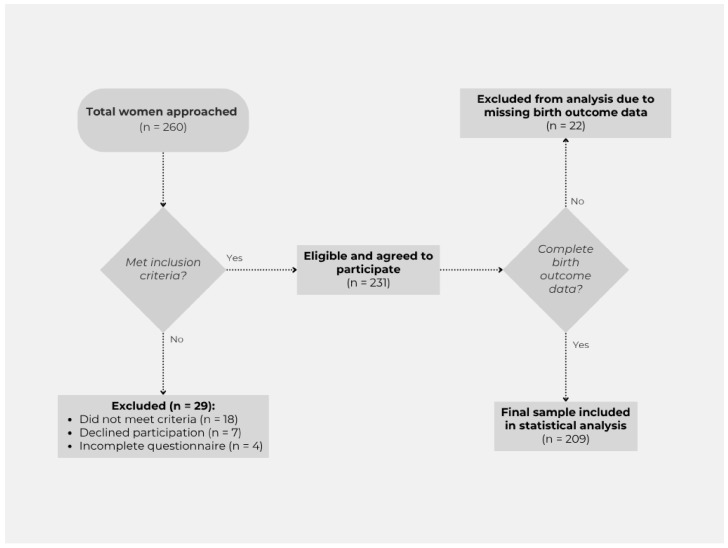
Flow diagram of participant recruitment and inclusion in the statistical analysis.

The study was carried out in a quantitative, cross-sectional research design in the autumn of 2024 in three segregated Roma communities in Szabolcs-Szatmár-Bereg County. The inclusion of Nyíregyháza, Tiszavasvári, and Nagyecsed in the research was based on the presence of well-defined, settlement-like segregated areas within the settlement structure and the concentrated nature of social and health disadvantages. In selecting the research sites, a key consideration was to reach communities where disadvantaged Roma women live in larger numbers and in relatively accessible locations, allowing for targeted data collection and exploration of relevant health behavioral characteristics. A peer sampling procedure was used for the study, considering spatial segregation, ethnic composition, and accessibility to research sites. Participants were recruited using a peer sampling (snowball) technique, involving trusted, locally active individuals such as members of the Roma self-government, family support center staff, and social workers. These peers identified and referred potential participants through their personal networks, which facilitated trust and participation. However, this method may have introduced bias, as recruitment was limited to the peers’ social networks, potentially leading to the over- or under-representation of certain subgroups. Women aged 18–65 years with permanent residence in one of the segregated communities included in the study were eligible for participation. Exclusion criteria included intellectual disability that would have prevented the completion of the questionnaire or the provision of informed consent. Participants were recruited in person by the researcher, with the assistance of the local Roma self-government, family support services, and social workers, either at participants’ homes or in community settings. No formal power analysis was conducted prior to data collection. Instead, the sample size was determined pragmatically, informed by the research team’s extensive prior experience in conducting long-term health sociology studies in the same settlements, and their in-depth knowledge of the local social and demographic context. Given the exploratory nature of the research and the absence of a comprehensive sampling frame for the marginalized Roma population, the final sample size reflected the maximum number of eligible and consenting women that could be reached during the study period. This approach ensured the feasibility of data collection while providing a sample size comparable to or larger than that of similar community-based studies among marginalized groups reported in the literature. Data collection was conducted in person using a paper-based questionnaire; no online or telephone interviews were conducted. No financial or other incentives were offered. A total of 260 women were approached, of whom 29 were excluded (18 did not meet the inclusion criteria, 7 declined participation, and 4 returned incomplete questionnaires). The remaining 231 women agreed to participate and completed the questionnaire. The final statistical analysis included data from 209 women, as birth outcome data were missing for 22 participants. Several questionnaire items were measured on a five-point Likert scale. The recruitment process and participant flow are illustrated in Figure 1.

The questionnaire consisted of 297 closed questions, which were compiled based on relevant national and international literature and the health priorities of Roma strategies. The questions covered socio-demographic characteristics (age, education, employment, housing), health behaviors (smoking, alcohol consumption, vitamin intake, substance use), participation in antenatal care, and birth outcomes, with a focus on the incidence of preterm birth, gestational week, and newborn status.

Data were statistically processed using IBM SPSS Statistics 25.0. Frequency distributions, means, and standard deviations were calculated to present the sample characteristics. The prevalence of preterm birth was analyzed by chi-square test along the main social, caregiving, and lifestyle variables. To explore the background factors in more depth, we also used a binary logistic regression model with maternal age, education, smoking and alcohol consumption habits, pregnancy planning, vitamin intake and antenatal care attendance as control variables. The significance level was set at *p* < 0.05 in all cases.

The study was conducted in full compliance with the relevant data protection and research ethics regulations. Participants were fully informed orally and in writing about the purpose of the research, data handling, and the voluntary and anonymous nature of the study. The study was granted ethics approval by the Scientific and Research Ethics Committee of the Health Sciences Council (TUKEB); TUKEB case no. BM/2120-1/2024.

## 3. Results

Of the 231 participants in the study, 42 women (18.2%) reported having had at least one pregnancy that ended in preterm birth—35 of these had experienced such an outcome once, while 7 had experienced it more than once.

Data on the smoking behavior of women who had experienced preterm birth during pregnancy showed that 21 (50%) smoked occasionally, 15 (35.7%) smoked regularly during the pregnancy, and 6 (14.3%) did not smoke. This suggests that nearly 86% of the women concerned were smoking in some form at the time of preterm delivery, confirming the importance of smoking as a risk factor. Smoking habits were recorded in three categories: non-smoker, occasional smoker (rarely, in social situations, a few times per month, with no more than 1–2 cigarettes on those occasions), and regular smoker (daily smoking, multiple cigarettes per day). In this study, the term “smoking in some form” referred exclusively to combustible tobacco products (manufactured or hand-rolled cigarettes); e-cigarettes and heated tobacco products were not assessed.

Alcohol consumption was also significant among women who had experienced preterm birth: 22 (64.7%) consumed alcohol occasionally, 1 (2.9%) consumed alcohol regularly, while 9 (26.5%) did not consume alcohol during pregnancy. This suggests that about two thirds of the women concerned had some alcohol consumption during pregnancy. Alcohol consumption was recorded in three categories: non-drinker, occasional drinker (1–2 times per month, during social or festive occasions, in small amounts—e.g., one glass of wine, one pint of beer, or one serving of spirits), and regular drinker (multiple times per week, several units of alcohol). International standard drink definitions were applied to quantify amounts.

Based on the questionnaire data, there were also significant differences in the frequency of antenatal care attendance among women who had experienced preterm birth: 13 (37.1%) attended antenatal care only a few times, 8 (22.9%) at least once a month, while 12 (34.3%) attended antenatal care every month. Only 1 person (2.9%) said that she had not attended any antenatal care. Although regular attendance reached one third, most responses indicate irregular or insufficient follow-up.

Regarding planning for pregnancy, 17 (40.5%) said that their pregnancy, which ended in premature birth, was a conscious decision, while 25 (59.5%) did not plan to have a child. This distribution supports the potential role of unintended pregnancy in the development of adverse perinatal outcomes.

These results are in line with findings in the national and international literature that smoking, alcohol consumption, unplanned childbearing, and inadequate antenatal care increase the risk of preterm birth. Table 1 presents the characteristics of women who experienced preterm birth. Multivariate analyses are used to explore the exact associations and are described later in the paper.

Table 2 presents the results of the multivariate logistic regression analysis of factors associated with preterm birth.

### Results of Multivariate Analysis

Vaginal infection during pregnancy—Associated with a fourfold higher risk of preterm birth (OR = 4.014, 95% CI: 1.576–10.222, *p* = 0.004; χ^2^ = 7.962, *p* = 0.005).

Drug use during pregnancy—Associated with a more than 25-fold higher risk of preterm birth (OR = 25.500, 95% CI: 2.876–226.125, *p* = 0.004; χ^2^ = 12.432, *p* < 0.001). The survey question on drug use recorded only the fact of use, without identifying the specific type of substance.

Alcohol consumption during pregnancy—Associated with nearly a fivefold higher risk of preterm birth (OR = 4.676, 95% CI: 2.165–10.098, *p* < 0.001; χ^2^ = 15.405, *p* < 0.001).

Smoking during pregnancy—Associated with almost a fivefold higher risk of preterm birth (OR = 4.783, 95% CI: 1.996–11.460, *p* < 0.001; χ^2^ = 12.770, *p* < 0.001).

To investigate the possible risk and protective factors underlying preterm birth, we also analyzed the role of folic acid/folate vitamin intake during pregnancy. The results showed a statistically significant, albeit weak, association between vitamin intake and the incidence of preterm birth (χ^2^(1) = 4.133, *p* = 0.042; Cramer’s V = 0.158). The rate of preterm birth was significantly lower among those who regularly took folic acid-containing prenatal vitamins (40.6% vs. 59.4%), suggesting a protective effect of vitamin intake.

A statistically proven association of moderate strength was also identified for drug use (χ^2^(1) = 17.252, *p* < 0.001; Cramer’s V = 0.302). Preterm birth was over twenty-two times more frequent among drug users (14.3% vs. 0.6%), highlighting the serious risk role of substance use.

**Figure 2:**
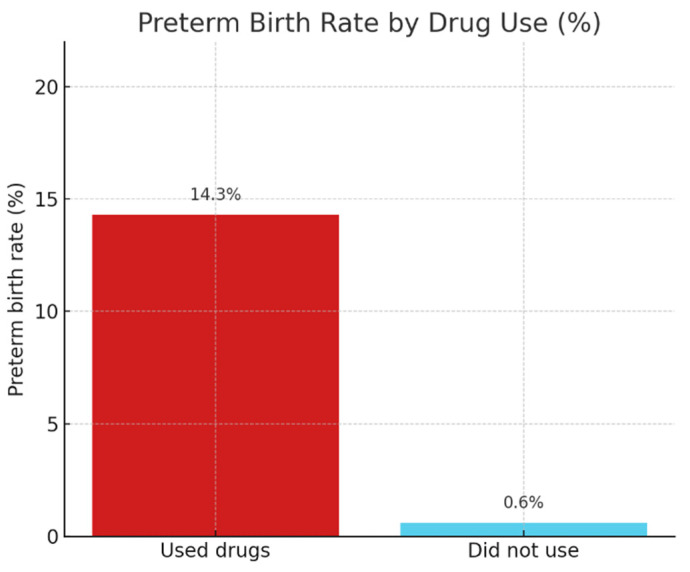
Preterm birth by drug use.

Figure 2 illustrates the rates of preterm births among drug-using and non-using pregnant women. It can be clearly seen that preterm birth was over twenty-two times more frequent among substance users, supporting the significant association found in the analysis (χ^2^(1) = 17.252; *p* < 0.001).

There was a medium-strength significant association between smoking and preterm birth (χ^2^(1) = 13.738; *p* < 0.001; Cramer’s V = 0.296). The prevalence of preterm birth was three times higher among women smokers (75.8%) compared to non-smokers (24.2%), confirming that smoking is one of the most prominent risk factors for perinatal outcomes.

**Figure 3:**
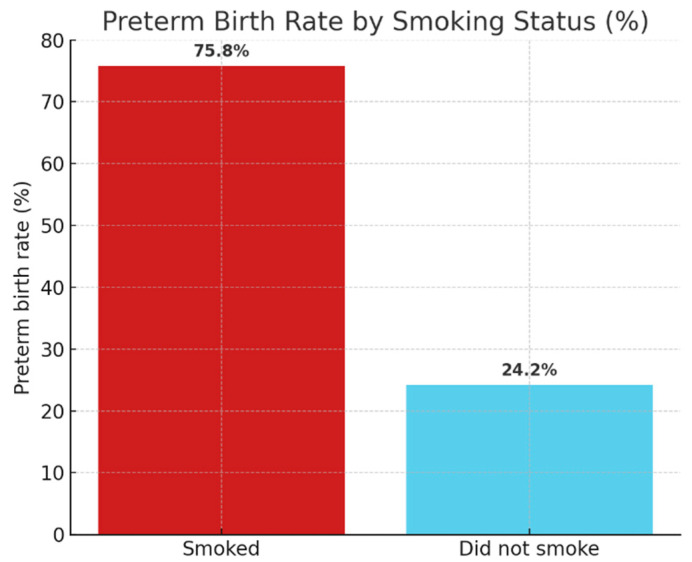
Preterm birth by smoking.

Figure 3 shows the rates of preterm births among smoking and non-smoking women. The incidence of preterm birth was found to be three times higher in pregnant women who smoked, confirming the statistically proven association (χ^2^(1) = 13.738; *p* < 0.001).

The effect of alcohol consumption was also found to be statistically significant (χ^2^(1) = 17.069; *p* < 0.001; Cramer’s V = 0.297). The rate of preterm births among pregnant women who consumed alcohol was 54.3% compared to 20.3% among non-drinkers—almost three times the risk.

**Figure 4:**
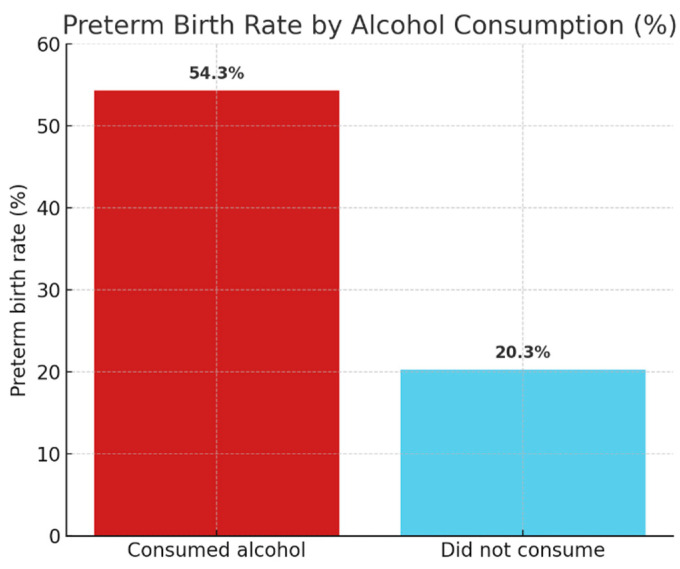
Preterm birth by alcohol use.

Figure 4 plots the prevalence of preterm births between alcohol-consuming and non-consuming pregnant women. The data show that alcohol consumption was associated with almost three times the risk, and the difference is significant (χ^2^(1) = 17.069; *p* < 0.001).

Finally, for the association between attendance at antenatal care and preterm birth, although the differences tended to be present (41.2% preterm birth rate among those who did not attend antenatal care compared to 23.9% among those who did), the association was not found to be statistically significant (χ^2^(1) = 2.699; *p* = 0.100; Cramer’s V = 0.125).

## 4. Discussion

The results of the present study confirm that the prevalence of preterm birth among Roma women living in segregated environments is not only related to biological but also to strongly social and lifestyle factors. Drug use emerged as one of the strongest predictors, carrying a more than 22-fold increased risk.

Drug use during pregnancy is a serious public health problem that can adversely affect both maternal and fetal outcomes. Available international data suggest that most psychoactive substances, including nicotine, alcohol, cannabis, cocaine, and opiates, can cross the placenta, directly harming the developing fetus. Numerous studies highlight that these exposures increase the risk of preterm birth, intrauterine growth retardation (SGA), low birth weight, and long-term neurocognitive impairment [6,7,12]. Cannabis and other drugs increase the likelihood of abnormalities affecting fetal brain development, while stimulants, particularly amphetamines and cocaine, can induce hypoxic conditions by impairing placental perfusion [23,24,25,26,27]. Prevention strategies can only be effective if they take into account social inequalities, gaps in access to reproductive health, and the complex needs of affected populations [20,28].

For a long time, pregnancy has acted as a preventive factor against psychoactive substance use, but current health experience in Szabolcs-Szatmár-Bereg County suggests that this protective effect has now been significantly weakened; the prevalence of drug use during pregnancy is on the rise, as our study shows, and has become a serious phenomenon requiring complex care and public health intervention in the region. The cross-sectional design of the study limits the ability to establish causal relationships between the identified risk factors and the occurrence of preterm birth. As data were collected at a single point in time, temporal sequences and potential changes over time could not be assessed.

## 5. Conclusions

We also found significant associations between smoking, alcohol consumption, and vaginal infections and preterm birth. Lack of informed pregnancy planning, and insufficient antenatal care attendance were also found to be risk factors, although not always statistically significant.

The protective effect of vitamin supplementation with folic acid/foliate in the prenatal period is suggested by the fact that regular vitamin supplementation was associated with lower rates of preterm birth, although this association was found to be weak. The evidence suggests that the development of preterm birth is a complex, multifaceted phenomenon in which social exposure, low health awareness, and inequalities in access play a role.

This study provides one of the most extensive analyses to date on preterm birth among women living in highly segregated Roma communities in Hungary, combining a large sample size with a comprehensive examination of sociodemographic, health behavior, and reproductive health indicators. The results of the study underline the urgent need for culturally specific, locally based prevention and health education programs in disadvantaged communities that can effectively target women at risk. Reducing smoking, alcohol, and substance abuse; encouraging involvement in antenatal care; and promoting awareness of childbearing can be key elements of such interventions.

Research can help to inform public health and social policy strategies that can reduce the incidence of preterm births and reduce perinatal health inequalities in Roma communities in the long term.

## Figures and Tables

**Table 1:** Characteristics of women who experienced preterm birth *(n* = 42).

Variable	Category	*n*	%
Smoking during pregnancy	Did not smoke	6	14.3
	Smoked occasionally	21	50.0
	Smoked regularly	15	35.7
Alcohol consumption during pregnancy	Did not consume	9	26.5
	Occasionally	22	64.7
	Regularly	1	2.9
Antenatal care attendance	None	1	2.9
	Only a few times	13	37.1
	At least once a month	8	22.9
	Every month	12	34.3
Pregnancy planning	Planned	17	40.5
	Unplanned	25	59.5

**Table 2:** Multivariate logistic regression analysis of factors associated with preterm birth.

Variable	OR	95% CI (Lower–Upper)	*p*-Value
Alcohol consumption during pregnancy	1.744	1.206–2.524	<0.01
Smoking during pregnancy	2.495	1.343–4.637	<0.01
Drug use during pregnancy	25.500	2.876–226.125	<0.001
Vitamin supplementation (folic acid)	0.448	0.204–0.982	<0.05
Vaginal infection during pregnancy	4.014	1.576–10.222	<0.01

## Data Availability

The data supporting the findings of this study are not publicly available due to privacy and ethical restrictions. However, anonymized data may be made available from the corresponding author upon reasonable request.
